# Relationship between ocular manifestations, laboratory findings, echocardiographic findings, and intravenous immunoglobulin resistance in Kawasaki disease

**DOI:** 10.1186/s12969-024-00985-1

**Published:** 2024-05-01

**Authors:** Mohsen Jari, Hajar Esmaeili

**Affiliations:** 1 Department of Pediatric Rheumatology, Imam Hossein Children’s Hospital, Isfahan University of Medical Sciences, Isfahan, Iran; 2 School of Medicine, Isfahan University of Medical Sciences, Isfahan, Iran

**Keywords:** Kawasaki disease, Ocular involvements, Uveitis, Children

## Abstract

**Background:**

This study investigates the incidence of ocular involvement in Kawasaki disease (KD) and evaluates the relationship between ocular manifestations, laboratory findings, echocardiographic findings, and intravenous immunoglobulin (IVIG) resistance.

**Methods:**

We conducted a cross-sectional study with 58 KD patients from June 2021 to March 2023. For all patients, a complete ophthalmologic examination and echocardiography were performed in the acute phase before starting the treatment. We analyzed the age, sex, mean of white blood cell (WBC) count, platelet count, erythrocyte sedimentation rate (ESR), C-reactive protein (CRP), levels of alanine aminotransferase (ALT) and aspartate aminotransferase (AST), echocardiographic findings and IVIG responses for all patients and compared the group with ocular involvement with the group without involvement.

**Results:**

The incidence of bilateral acute conjunctivitis was 70.7%, while that of acute uveitis was 30%. Patients with uveitis had significantly higher rates of Coronary artery dilatation and IVIG resistance, as well as higher mean levels of WBC, platelet, and CRP compared to those without uveitis. (*P* < 0.05). Additionally, the age of patients with uveitis involvement was lower than those without involvement. No significant relationships existed between ESR, AST, or ALT values and uveitis (*P* > 0.05). Furthermore, no significant correlations existed between any examined items and acute bilateral conjunctivitis.

**Conclusion:**

Uveitis in KD is significantly associated with coronary artery dilatation, IVIG resistance, higher WBC count, platelet count, and CRP level.

## Background

Henoch Schönlein purpura (HSP) and Kawasaki disease (KD) are the most common types of childhood vasculitis [1]. KD is a small to medium vessel vasculitis that affects infants and young children [2]. Clinical diagnosis is based on guidelines provided by the American Heart Association (AHA), which states that a fever for more than five days plus at least four clinical signs not explained by another disease can confirm the diagnosis of KD. These signs include oropharyngeal mucous membrane changes, bilateral bulbar conjunctival injection, polymorphous rash, changes in the peripheral extremities, and cervical lymphadenopathy [3]. KD can present various ophthalmic manifestations, including bilateral conjunctivitis, uveitis, iridocyclitis, superficial punctate keratitis, vitreous opacities, and papilledema [4].

Previous studies have shown that bilateral non-exudative bulbar conjunctivitis and acute uveitis are seen in about 80 − 90% and 20 − 80% of the KD patients, respectively [4–9]. Burns et al. reported an 83%incidence rate of anterior uveitis among patients who underwent examination during the first week of their illness [10].

Cardiovascular complications are the primary cause of morbidity and mortality associated with KD, both during the acute illness and in the long term [3]. Incomplete KD is associated with delayed diagnosis and treatment, which in turn can lead to the development of coronary artery lesions (CALs) [7, 11].

A few studies have reported the prevalence of ocular involvement in KD, and they have not sufficiently discussed the relationship between ocular involvement and laboratory findings or coronary artery involvement. In one study, uveitis was associated with coronary artery dilatation, higher neutrophil count, and higher CRP levels in children with KD [12].

This study aimed to investigate the incidence of ocular complications, grading of uveitis, and the relationship between these complications with sex, age, laboratory data, coronary artery dilatation, and IVIG resistance in KD.

## Materials and methods

This study is a cross-sectional study of patients with KD from June 2021 to March 2023. There were 58 patients with KD studied. KD was diagnosed by a pediatric rheumatologist based on AHA criteria. All patients underwent serological testing. Any cases that tested positive for COVID-19 by PCR were excluded from the study. An experienced ophthalmologist examined all patients at admission and three months later. Ophthalmological examinations were performed in the acute phase before the standard treatment and included slit lamp biomicroscopy and fundoscopy. Uveitis was diagnosed and graded using the Standardization of Uveitis Nomenclature grading scheme for anterior chamber cells and flare [13, 14].

The diagnosis of coronary artery dilation in KD was based on the Japanese Ministry of Health (JPH) criteria. According to the JPH criteria, a dilated coronary artery is considered when the internal lumen diameter is greater than 3 mm for children younger than five years old or more significant than 4 mm in children at least five years old [3].

All patients were treated with IVIG (2 g/kg), aspirin (80–100 mg/kg) as the first-line treatment, and then aspirin 3–5 mg/kg/day as a daily dose. In patients who showed resistance to IVIG (persistence of fever or recurrence of fever 36 h after IVIG infusion), a second dose of IVIG (2 g/kg) was administered.

Age, sex, laboratory values, including WBC, platelet count, ESR, CRP levels of AST and ALT, and echocardiographic findings were collected and recorded.

The collected data were entered into SPSS software version 26. The data were presented as n (%) or mean ± standard deviation (SD). At the level of inferential statistics, an independent t-test and chi-score test were used to compare the mean of quantitative variables and the frequency distribution of qualitative variables between two types of ocular involvement. *P* values of < 0.05 were considered statistically significant.

## Results

The present study, 58 children were studied, 27 (46.6%) girls and 31 (53.4%) boys. The average age of the children was 47.59 ± 27.05 months. In the ocular examination, 41 cases (70.7%) had bilateral conjunctivitis. Acute anterior uveitis was unilateral in two cases (3.4%) and bilateral in 14 cases (24.1%). Also, two patients (3.4%) of patients had acute, intermediate uveitis, which was unilateral (Table 1).

**Table 1:** Frequency percentage of ocular involvement in different parts of the affected eye in Kawasaki disease patients

Characteristic	Unilateral involvement *n* (%)	Bilateral involvement *n* (%)
**Conjunctivitis**	0 (0%)	41(70.7%)
**Scleral or conjunctival scarring**	0 (0%)	0 (0%)
**Acute anterior uveitis**	2(3.4%)	14(24.1%)
**Acute intermediate Uveitis**	2(3.4%)	0 (0%)

Among 18 patients with uveitis, 27.8% had grade 1, 55.6% had grade 2, 11.1% had grade 3, and 5.6% had grade 4 uveitis (Table 2).

**Table 2:** The Standardization of Uveitis Nomenclature (SUN) grading scheme for uveitis and ocular findings in Kawasaki disease patients with uveitis

Grade	No. of cells	No. of patients (%)	Stage
**0**	< 1	0 (0%)	-
**0.5+**	1–5	0 (0%)	-
**1+**	6–15	5(27.8%)	Acute
**2+**	16–25	10(55.6%)	Acute
**3+**	26–50	2(11.1%)	Acute
**4+**	> 50	1(5.6%)	Acute
		Total 18(100%)	

In investigating the basic and clinical characteristics of patients with and without conjunctivitis, it was found that the number of boys in the group with conjunctivitis and the group without conjunctivitis was more than that of girls. The average age of children in the group with conjunctivitis was 45.19 ± 25.84 months, and in the group without conjunctivitis was 53.35 ± 29.78 months, which was less in the group with conjunctivitis than the group without conjunctivitis. However, the difference in age and sex between the two groups was not significant (*P* value > 0.05). In addition, the average of laboratory tests such as WBC, platelets, ESR, CRP, ALT, and AST were not significantly different between the two groups (*P* value > 0.05). Coronary artery dilation was 9.8% in the group with conjunctivitis and 17.6% in the group without conjunctivitis, which was more significant in the group without conjunctivitis. IVIG resistance was 9.8% in the group with conjunctivitis and 11.8% in the group without conjunctivitis, which was more in the group without conjunctivitis. However, these differences were not statistically significant (*P* value > 0.05). (Table 3).

**Table 3:** Comparative investigation of basic and laboratory characteristics of Kawasaki disease patients according to the involvement of conjunctivitis

Variables		Conjunctivitis (-) group (*n* = 17)	Conjunctivitis (+) group (*n* = 41)	*P* value
**Sex**	**Female**	7(41.2%)	20(48.8%)	0.773
	**Male**	10(58.8%)	21(51.2%)	
**Age; month**		53.35 ± 29.78	45.19 ± 25.84	0.300
**WBC; ×10** ^**9**^ **/L**		13.82 ± 10.07	14.19 ± 9.50	0.765
**Platelets; ×10** ^**9**^ **/L**		480.88 ± 119.51	532.63 ± 217.39	0.398
**ESR; mm/hr**		63.76 ± 12.45	63.56 ± 18.58	0.967
**CRP; mg/l**		33.06 ± 22.07	45.88 ± 33.81	0.156
**ALT; IU/l**		40.06 ± 15.92	40.61 ± 14.88	0.903
**AST; IU/l**		39.37 ± 22.60	34.73 ± 13.08	0.335
**Coronary artery dilatation**		3(17.6%)	4(9.8%)	0.407
**IVIG resistance**		2(11.8%)	4(9.8%)	0.819

In investigating the basic and clinical characteristics of patients with and without uveitis, it was found that there was no significant difference in the frequency distribution of children’s gender between the two groups (*P* value > 0.05). The average age of children in the group with uveitis was 25.28 ± 9.78 months, and in the group without uveitis was 57.62 ± 26.35 months, which means the age in the group with uveitis was significantly lower than the group without involvement (*P* value < 0.001). The mean of WBC in the group with uveitis was 17.93 ± 4.39 × 10^9/L, and in the group without uveitis was 12.35 ± 9.19 × 10^9/L. The mean of platelets in the group with uveitis was 755.56 ± 147.94 × 10^9/L and in the group without uveitis was 410.32 ± 130.39 × 10^9/L and the mean of CRP in the group with uveitis was 83.5 ± 15.31 mg/L and in the group without uveitis was 23.5 ± 13.22 mg/l, that means the mean of WBC, platelets, and CRP in the group with uveitis was significantly higher than the group without such involvement (*P* value < 0.05). However, the mean of ESR and liver factors (ALT, AST) was not significantly different between the two groups (*P* value > 0.05). Coronary artery dilation was 27.8% in the group with uveitis and 0.5% in the group without uveitis, and IVIG resistance was 22.2% in the group with uveitis and 0.5% in the group without uveitis٫ That means coronary artery dilation and IVIG resistance were significantly higher in the group with uveitis(*P* value < 0.05) (Table 4).

**Table 4:** Comparative investigation of basic and laboratory characteristics of Kawasaki disease patients according to the involvement of uveitis

Variables		Uveitis (-) group (*n* = 40)	Uveitis (+) group (*n* = 18)	*P* value
**Sex**	**Female**	17(42.5%)	10(55.6%)	0.356
	**Male**	23(57.5%)	8(44.4%)	
**Age; month**		57.62 ± 26.35	25.28 ± 9.78	< 0.001
**WBC; ×10** ^**9**^ **/L**		12.35 ± 9.19	17.93 ± 4.39	< 0.001
**Platelets; ×10** ^**9**^ **/L**		410.32 ± 130.39	755.56 ± 147.94	0.116
**ESR; mm/hr**		66.62 ± 16.94	56.94 ± 15.19	0.043
**CRP; mg/l**		23.50 ± 13.22	83.50 ± 15.31	< 0.001
**ALT; IU/l**		40.61 ± 16.13	40.11 ± 12.74	0.908
**AST; IU/l**		35.43 ± 18.30	37.33 ± 10.59	0.685
**Coronary artery dilatation**		2(5.0%)	5(27.8%)	0.025
**IVIG resistance**		2(5%)	4(22.2%)	0.046

## Discussion

The present study showed that more than two-thirds of KD patients presented with bilateral conjunctivitis in the acute phase (first two weeks), and less than one-third of the patients presented with uveitis in the acute phase. No other ocular manifestations were observed in this study. Regarding the anatomical involvement of KD uveitis, the anterior segment was the most common part. In uveitis sufferers, more than half of them showed grade 2 + uveitis. Grade 1 + uveitis had the most patients after that. In the current study, the incidence of acute bilateral conjunctivitis was 70.7%, similar to previous studies. Previous studies reported this prevalence between 60 and 90% [12, 15–18].

Choi et al. studied 110 cases of KD. In their study, 29% of the patients had acute uveitis in the acute phase of KD. Most cases of uveitis in their research were graded 1 + and 2+. In addition, in their study, age, CRP, ESR, and neutrophil count were higher in patients with uveitis than those without uveitis. Coronary artery dilatation and response to IVIG treatment were not significantly different between those without uveitis involvement [15]. Also, Shiari et al. examined 36 patients with KD and found that about one-third of the patients in their study had acute uveitis. Most of the uveitis cases in their study were graded as 1 + and 2+, similar to the Choi et al. study. In addition, according to their research, uveitis is associated with coronary artery dilatation, higher neutrophil count, and higher CRP levels. In the study of Shiari et al., acute bilateral non-exudative conjunctivitis and acute uveitis had no significant relationship with age and sex [12].

In the present study, acute bilateral conjunctivitis did not show a significant relationship with any of the investigated items, i.e., sex, age, laboratory findings, coronary artery dilation, and resistance to IVIG. The present study showed that the age in the group with uveitis involvement was significantly lower than in the group without uveitis involvement, which contradicts the findings of Choi et al.’s study. Their study showed that the age of uveitis patients is significantly higher than that of non-uveitis patients [15]. Sample size and genetic and environmental factors may be the reason for differences in studies.

Also, the present study showed that the mean of WBC, platelet, and CRP in the group with uveitis involvement was higher than that of the group without involvement.

In the study of Shiari et al., uveitis was directly related to the higher level of CRP. In that study, the average WBC in the group with involvement of uveitis was higher than the group without involvement, and the average platelet in the group without involvement was higher than the group with uveitis. Still, these results were not statistically significant [12].

In Choi et al.’s study, the levels of CRP, ESR, WBC and platelet in the group with uveitis were higher than in patients with KD without uveitis. However, the findings were not statistically significant [15].

The present study showed a statistically significant relationship between acute uveitis and coronary artery dilation, which was also reported in the study by Shiari et al. But in Choi et al.’s study, there was no significant difference in coronary artery dilation between uveitis and non-uveitis groups [15]. Also, according to the results of Lee et al.’s study, the risk of CALs in uveitis patients with incomplete KD is lower [16]. These results are not similar to the results of the present study. The present study showed that the resistance to IVIG was higher in the group with uveitis involvement than in the group without involvement. In Choi et al.’s study, there was no statistical difference in the response to IVIG in the control and uveitis groups. Still, the response to the first IVIG was higher in the control group compared to the uveitis group [15]. In the study of Lee et al. IVIG-resistant KD was treated using additional IVIG infusions or second-line therapy, such as systemic corticosteroids or infliximab. The necessity of second-line treatment, such as systemic corticosteroid or infliximab, was significantly higher in the group without acute uveitis compared to the group with acute uveitis. This result is not consistent with the present study. It is worth noting that in the study of Lee et al., all patients with KD were not included, and only those with suspected IKD underwent ophthalmologic examinations [16].

Coronary artery aneurysm is one of the most dangerous and important complications of KD. Early diagnosis and treatment can prevent this complication, which can be life-saving. Also, many studies have shown that patients who are resistant to initial IVIG are at increased risk of developing coronary artery abnormalities [3]. This highlights the importance of the relationship between uveitis and resistance to IVIG. It can be contended that acute uveitis may be considered one of the essential diagnostic criteria for KD.

In our study, ophthalmological examinations were performed for all patients with KD, which can be considered the strength of this study. However, our study had limitations, such as a small sample size and a single institution. Also, we did not study the relationship of some factors, such as the duration of fever, the neutrophils/ lymphocytes ratio, albumin, and sodium.

Conclusion: We found that acute uveitis has a significant relationship with coronary artery dilatation and IVIG resistance in KD. Based on this result, our recommendation is that all KD patients consult with an ophthalmologist.

## Data Availability

The data are available on request to the corresponding author.

## Abbreviations

KD: Kawasaki disease
IVIG: intravenous immunoglobulin
WBC: White blood cell count
ESR: Erythrocyte sedimentation rate
CRP: C-reactive protein
ALT: Alanine aminotransferase
AST: Aspartate aminotransferase
CALs: Coronary artery lesions

## References

[1] Pamela F, Weiss (2012). Pediatric Vasculitis. Pediatr Clin North Am.

[2] Singh S, Vignesh P, Burgner D (2015). The epidemiology of Kawasaki disease: a global update. Arch Dis Child.

[3] McCrindle BW, Rowley AH, Newburger JW (2017). Diagnosis, treatment, and long-term management of Kawasaki disease. A scientific statement for health professionals from the American Heart Association. Circulation.

[4] Ohno S, Miyajima T, Higuchi M (1982). Ocular manifestations of Kawasaki’s disease (mucocutaneous lymph node syndrome). Am J Ophthalmol.

[5] Burke MJ, Rennebohm RM (1981). Eye involvement in Kawasaki dis- ease. J Pediatr Ophthalmol Strabismus.

[6] Burke MJ, Rennebohm RM, Crowe W, Levinson JE (1981). Follow-up ophthalmologic examinations in children with Kawasaki’s dis- ease. Am J Ophthalmol.

[7] Freeman AF, Shulman ST (2006). Kawasaki disease: summary of the American Heart Association guidelines. Am Fam Physician.

[8] Madhusudan S, Singh S, Suri D, Gupta A, Gupta A (2014). Acute anterior uveitis as the presenting feature of Kawasaki disease. Indian J Pediatr.

[9] Cunningham ET (2000). Uveitis in children. Ocul Immunol Inflamm.

[10] Burns JC, Joffe L, Sargent RA, Glode MP (1985). Anterior uveitis associated with Kawasaki syndrome. Pediatr Infect Dis.

[11] Daisuke Sudo Y, Monobe M, Yashiro (2012). Coronary artery lesions of incomplete Kawasaki disease: a nationwide survey in Japan. Eur J Pediatr.

[12] Shiari R, Jari M, Karimi S (2020). Relationship between ocular involvement and clinical manifestations, laboratory findings, and coronary artery dilatation in Kawasaki disease. Eye (Lond).

[13] Guney E, Tugal-Tutkun I (2013). Symptoms and signs of anterior uveitis. US Ophthalmic Rev.

[14] Jabs DA, Nussenblatt RB, Rosenbaum JT. Standardization of Uveitis Nomenclature (SUN) Working Group. Standardization of uveitis nomenclature for reporting clinical data. Results of the First International Workshop. Am J Ophthalmol. 2005;140: 509–16. 10.1016/j.ajo.2005.03.057 PMC8935739 16196117

[15] Choi HS, Lee SB, Kwon JH (2015). Uveitis as an important ocular sign to help early diagnosis in Kawasaki disease. Korean J Pdiatr.

[16] Lee KJ, Kim HJ, Kim MJ (2016). Usefulness of anterior uveitis as an additional tool for diagnosing incomplete Kawasaki disease. Korean J Pediatr.

[17] Jacob JL, Polomeno RC, Chad Z, Lapointe N (1982). Ocular manifes- tations of Kawasaki disease (mucocutaneous lymph node syndrome). Can J Ophthalmol.

[18] Ohno S, Miyajima T, Higuchi M (1982). Ocular manifestations of Kawasaki’s disease (mucocutaneous lymph node syndrome). Am J Ophthalmol.

